# Kidney Disease-Specific Quality of Life among Patients on Hemodialysis

**DOI:** 10.1155/2021/8876559

**Published:** 2021-04-07

**Authors:** Issa Al Salmi, Pramod Kamble, Eilean Rathinasamy Lazarus, Melba Sheila D'Souza, Yaqoob Al Maimani, Suad Hannawi

**Affiliations:** ^1^The Royal Hospital, 23 July Street, P. O. Box 1331, Code 111, Muscat, Oman; ^2^Senior Specialist Nephrologist, Royal Hospital Ministry of Health, Muscat, Oman; ^3^College of Nursing, Sultan Qaboos University, Muscat, Oman; ^4^School of Nursing, Thompson Rivers University, Kamloops, Canada; ^5^Senior Nephrologist & Superintendent, Bowsher Dialysis Unit, Muscat, Oman; ^6^Medicine Department, Mohap, Dubai, UAE

## Abstract

**Introduction:**

Quality of life (QoL) of hemodialysis patients can be examined in two aspects: kidney-specific quality of life and general quality of life.

**Objective:**

To determine the QoL among patients undergoing hemodialysis, to assess patients' QoL on hemodialysis, and to determine the factors associated with QoL among hemodialysis patients in Oman.

**Method:**

A cross-sectional study was carried out with 205 patients to measure the QoL across various demographic and clinical variables in Oman. The Arabic version of the KDQOL-SFtool was used to collect data from patients undergoing hemodialysis to give QoL quantitative measures.

**Results:**

The physical-QoL was 45.7 (95% CI, 44.3, 47.0), which is less than half that of a healthy human. The emotional-QoL is 53.33 (95% CI, 51.1, 55.5), slightly more than half in a healthy human-QoL. The difference between physical and emotional-QoL scores is −7.66 (95% CI, −10.3, -5.1), showing that physical QoL is significantly less than emotional-QoL. The overall general QoL score was 49.5 (95% CI, 47.8, 51.2), half the QoL score of a healthy human. Younger patients are also more likely to experience emotional problems compared with older patients. Patients with 5–8 mg/l levels of serum creatinine have lower emotional wellbeing. People on low incomes experienced social difficulties, while the maximum burden was found in physical activities and minimum social function.

**Conclusion:**

Both physical (45.7) and emotional (53.3) QoL scores in dialysis patients are nearly half those of an average human. Hence, there is a poor QoL among dialysis patients like other studies, and therefore, further improvement of renal rehabilitation in dialysis patients is warranted to improve patients' QoL.

## 1. Introduction

Worldwide, the incidence and prevalence of End-Stage Kidney Disease (ESKD) are increasing in both developed and developing countries (National Kidney Foundation, 2002) [1]. In Oman, this increase is attributable to the growing burden of noncommunicable diseases (NCD). Oman, one of six countries that make up the Gulf region, is faced with an epidemic of chronic NCD, including diabetes and hypertension [2,3]. Also, few studies have been conducted in Oman that provides evidence of kidney dysfunction's prevalence and incidence. A systematic review of the incidence and prevalence of kidney dysfunction cases in the Gulf Cooperation Council (GCC) found a 38% prevalence of ESKD among diabetic patients [4].

Globally, there is a 30% increase in the prevalence of chronic kidney disease (CKD) among diabetic patients that progresses to cardiovascular disease (American Diabetes Association, 2002; World Life Expectancy, 2015). Numerous studies have concluded that Diabetes is the leading cause of ESKD [5]. A study conducted in the GCC region found that ESKD was present in 30% of diabetic patients in Bahrain, 14.5% in Oman, and 60% in Saudi Arabia [2, 6, 7]. Also, diabetes mellitus patients are at increased risk of complications that can lead to CKD, eventually leading to poor health, a deterioration in life expectancy, and a decreased QoL. Furthermore, increased hospitalizations among individuals with ESKD can lead to a drain on healthcare resources, thus causing financial stress [7].

Statistics have shown that CKD deaths in Oman account for 3.32% of total deaths per year, with the age-adjusted death rate estimated at 25.79 per 100,000 population (WHO, 2011). There is considerable evidence that ESKD is responsible for high levels of disability in various aspects of people's lives, leading to impaired QoL. The availability of various renal replacement therapies (RRT) is known to reduce the severity of symptoms, thus increasing life expectancy among ESKD patients [8, 9]. Unfortunately, hemodialysis therapy is time-intensive, expensive, and requires fluid and dietary restrictions [9–11]. Furthermore, long-term dialysis therapy can have a negative effect on caregivers through the loss of freedom, increased dependence, disruption of marital and family social life, and reduced financial circumstances, thereby leading to significant disruptions in the lifestyles of both patients and their families [11]. Additionally, other significant areas of life affected by ESKD and its treatment include but are not limited to disruption in employment, eating habits, vacation activities, sense of security, self-esteem, social relationships, and the inability to enjoy life due to physical, psychological, socioeconomic, and environmental aspects of their lives being negatively affected [12, 13].

Most patients suffering from ESKD will need hemodialysis two or three times a week. The commitment from both the patient and their caregivers, who in many cases will be a family member, is considerable [13]. Both the patient and caregiver are negatively burdened, and the caregiver's QoL may also be significantly affected significantly [14].

Additionally, patients and their caregivers may suffer functional and cognitive impairments, with primary caregivers also having to prepare special diets and provide their medication [15].

According to the Oman Ministry of Health [MOH] (2013), kidney dysfunction cases are increasing, and the country needs to reduce its health expenditure estimated by approximately 10–14 million USD per year to afford RRT. Although Omani citizens currently have access to free healthcare, this increased expenditure will soon affect healthcare resource distribution [16]. A concerted effort to address ESKD-related health expenditure is imperative, first of all, by assessing the QoL among hemodialysis patients. QoL of patients undergoing hemodialysis decreases in the various stages of kidney disease; this may be attributed to sociodemographic and clinical risk factors in the person. Patient-reported outcomes like QoL of patients are underestimated when providing medical care, which is an integral part of assessment and the impact of quality care. Hence, we aim to determine the QoL among patients undergoing hemodialysis to assess patients' QoL on hemodialysis and determine the factors associated with QoL among hemodialysis patients in Oman.

## 2. Methodology

### 2.1. Research Design

A cross-sectional study design has been adopted, which uses a standardized, short-form Kidney Disease Quality of Life (KDQOL) questionnaire, of which Arabic Version 1.3 (KDQOL-SF) is developed [17, 18].

### 2.2. Setting

The study was conducted at the Bowsher Dialysis Unit (BDU) at the Royal Hospital in Muscat, Oman. The Sultanate of Oman is administratively divided into four governorates and five regions. The capital city is Muscat, where the Bowsher Polyclinic is located (Ministry of Information, 2002). Most of the people in Oman can access dialysis units that are nearer to them. The BDU is a multispecialty polyclinic, with 42 high-tech dialysis units operating 24 hours a day.

### 2.3. Sample Size

The required sample size for this study was calculated based on the following equation:

(1) $$\begin{array}{c} n=\frac{z^{2}p\left(1-p\right)}{\varepsilon^{2}}, \end{array}$$

where *n* = sample size, *z* = value for the *z* distribution (1.96) based on the desired confidence level (95%), and *p* = the estimated proportion of a parameter from earlier studies. In this case, the proportion of the population falls into a certain category that needs to be estimated. For this type of study, a value of 14.5% is recommended (Atta, 2008), *ε*  = error of the estimate, which was assumed to be 0.05:

(2) $$\begin{array}{c} n=\frac{1.96^{2}\times 0.14\left(1-0.14\right)}{0.05^{2}}=185. \end{array}$$

Allowing for a 10% rate of attrition, a sample of 205 patients was decided upon.

### 2.4. Sampling Technique

A convenience sampling technique was employed to select the study participants. Patients receiving hemodialysis were recruited using the following inclusion and exclusion criteria.

## 3. Eligibility Criteria for Selection of Samples

### 3.1. Inclusion Criteria

- Patients receiving hemodialysis regularly
- Patients receiving hemodialysis for more than six weeks
- Patients between 30 and 60 years old (Amal et al., 2008)
- Patients able to understand either English or Arabic
- Patients willing to participate in the study

### 3.2. Exclusion Criteria

- Patients who are known to have a psychiatric disorder
- Patients who are undergoing hemodialysis for a condition other than CKD
- Patient with an altered level of consciousness
- Very sick patients
- Patients who are having hemodialysis having rejected a kidney transplant
- Patients with an acute kidney injury

## 4. Study Instruments and Description

### 4.1. Demographic Variables

The demographic variables consist of age, gender, educational status, occupation, marital status, place of residence, type of family, number of family members, family monthly income, and distance of residence from the nearest health center/hospital.

### 4.2. Clinical Variables

The clinical variables include the presence of comorbidity, the duration that they have been receiving RRT, the frequency of hemodialysis per week, physical activity, hemoglobin levels, serum creatinine levels, and BMI.

## 5. Measurement Tool 1: Kidney Disease Quality of Life (KDQOL)

The KDQOL-SF 1.3 standardized tool in Arabic version was developed by Samar Abd ElHafeez and has been used to assess QoL among hemodialysis patients. This KDQOL-SF tool [18] has been checked for validity and reliability (*r* = 0.78–0.92). The tool will be used to assess the QoL among patients receiving hemodialysis in Oman. This tool includes the effect of kidney disease on daily life and the satisfaction of care received by patients undergoing hemodialysis. The Kidney Disease Quality of Life (KDQOL) questionnaire, short-form, version 1.3 (KDQOL-SF), consists of 36 questions, 35 of which are compressed into eight multi-item scales. Also, the tool will be modified to include other variables to be assessed as follows.

The SF36 has two subscales summarized into physical health and mental health dimensions, as shown in Table 1.

## 6. Scoring Procedure and Interpretation

A score between 0 and 100 is calculated based on well-defined guidelines, with a higher score indicating a better health state. Each item is put on a 0–100 range, with the lowest and highest possible scores set at 0 and 100, respectively. The scores represent the percentage of total possible scores achieved [18]. More instructions are provided on the main document as needed. The Arabic (KDQOL-SF) version 1.3 is also available from RAND and the University of Arizona (Supplemental figure-online). The KDQOL-SF^TM^ tool was translated from English to Arabic and backtranslated by a professional translator for consistency and validity. The scoring was uniform across English and Arabic versions.

## 7. Pilot Study

The researchers conducted a pilot study using 10% of the sample group (21 participants) using the translated Arabic version to ascertain the tool's cultural adaptability and its reliability and validity. The participants who participated in the pilot study were then excluded from the main study. The results showed that no further modifications needed to be made.

## 8. Data Collection and Ethical Clearance

Permission was obtained to conduct this study in its dialysis unit, with ethical approval from the Research Ethics Committee at the College of Nursing and the Royal Hospital. Eligible participants were identified with the assistance of the Omani staff nurses in the dialysis units and informed consent, which detailed the purpose and benefits of study participation. All the data collected from the patients by the research assistants were confidential and anonymous. Confidentiality has also been maintained throughout the study.

## 9. Statistical Analysis

To examine the data normality of the continuous data and distribution of the sample, we used Kolmogorov-Smirnov test for a sample size of more than 50. When *p* < 0.05, null hypothesis is accepted, the data are called normally distributed. For this medium-sized sample of 205, at absolute *z* value +3.29, the distribution of the sample was normal. Hemoglobin levels, serum creatinine levels, and BMI followed a normal distribution and was expressed as mean and standard deviation. We had 205 sample size, with *Z*-scores −1.81, at level of significance *p* < 0.001. The data showed a normal distribution.

In order to answer the research questions, descriptive and inferential statistics were used on all the variables. To confirm the measurements' consistency, Cronbach's alpha score was calculated and tested for each of the measuring variables. Descriptive analyses were carried out to explain the demographic variables, and ANOVA and pooled *t* tests were performed to make inferences. The statistical software programs Minitab 17.0 and SPSS 16.0 were used for the data analysis.

## 10. Results

### 10.1. Demographic Findings

A total of 205 patients were included in this study, and no dropouts were recorded. 37% of the patients were aged between 40 and 49 years, 54% of the patients were male, and 43% of the patients were only educated up to primary school level or had no formal education, which is a significant issue in considering management choices. A total of 72% of the patients were married, and 19% were divorced or widowed. 80% of the patients were in the employment category, and 87% made more than 4000 USD per year. A total of 85% of the patients live in an urban area, while 47% of them are from a traditional nuclear family. A total of 44.9% of the patients had diabetes mellitus, and 79.5% had been dialyzed for more than six months. It was found that 76.6% of the patients have dialysis more than twice a week, which shows its seriousness. A total of 52.2% reported 6–8.5 mg/dl of hemoglobin, and 41.5% had more than 9 mg/dl of hemoglobin. The patients' serum creatinine level shows that 78.5% had a level of 20–40 mg/dl, and a majority (59.5%) of patients were underweight.

**Figure 1:**
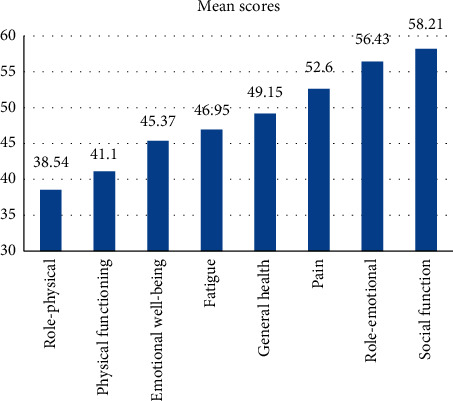
Level of burden in each item of quality of life.

Figure 1 shows the level of burden in each type of QoL category for hemodialysis patients. The highest burden was found for physical activity, while the lowest was found in social function.

Assuming each measure has the same weight, the overall physical QoL in this study was 45.67 (95% CI, 44.291, 47.041), less than half than that of a healthy human. The physical functioning and physical roles are significantly less than the other three measures (*p* < 0.01). Physical functioning and physical roles do not differ significantly (*p* > 0.52), and the other three measures do not differ significantly, either (*p* > 0.25). The means and the standard deviations of the measures are given below, and the means of the measures of physical QoL are summarized in Table 2.

Assuming each measure has the same weight, the overall emotional QoL in this study was 53.33 (95% CI, 51.1, 55.5) which is slightly more than half that in a healthy human QoL. The emotional QoL was significantly worse than the other two measures (*p* < 0.01) although the other two measures do not differ significantly (*p* > 0.31). The means and the standard deviations of the measures are given below, while the means of the measures of emotional QoL are summarized in Table 3.

There was a significant effect of the patient's QoL physical functioning with older age (*p* < 0.05), income (*p* < 0.04), and frequency of dialysis (*p* < 0.03) (Table 4). There was a higher significance of patent's QoL role physical with education (*p* < 0.01) and diabetes mellitus (*p* < 0.04). There is a positive relationship between patient's pain with older age (*p* < 0.03) and frequency of dialysis (*p* < 0.05). There is positive QoL of general health with age (*p* < 0.01), diabetes mellitus (*p* < 0.02), and frequency of dialysis (*p* < 0.03). There was significant relationship between QoL fatigue and age (*p* < 0.05), education (*p* < 0.03), and duration of dialysis (*p* < 0.04).

### 10.2. Correlation between Physical and Emotional Dimensions of QoL

**Figure 2:**
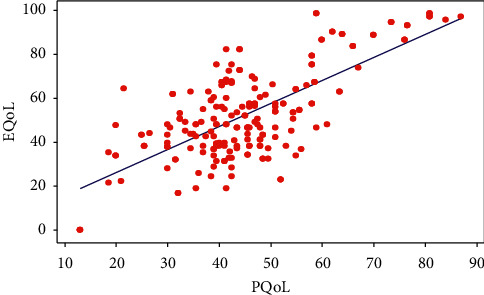
Scatter diagram of the relationship between physical and emotional qualities of life.

The difference between the physical and emotional QoL score is −7.66 (95% CI, −10.3, −5.1) which shows that the physical QoL is significantly worse than the emotional QoL.  Figure 2 shows the relationship between the physical QoL and emotional QoL. A linear relationship was observed between these measures, which can be given in the following linear equation with *R* square 49%. Emotional QoL = 5.32 + 1.05 physical QoL.

## 11. Discussion

The largest proportion (37%) of the patients was between 40 and 49 years old. In addition, 54% of the patients were male, 43% were not educated or educated only up to the primary level, while 72% were married. 80% of the patients were in employment and the largest proportion (87%) of patients' income was more significant than 3,900 USD, but it must be looked for some more categories. A total of 44.9% of the patients had diabetes mellitus, and 59.6% were underweight. Most of the demographic and clinical variables are associated with QoL measures.

The patients with cardiac disease and underweight patients had low quality of life and low physical functioning. Young people, educated people, widowed people, and people on low incomes group have problems carrying out their physical roles. The educated groups were much affected than the noneducated or primary educated group regarding their physical role.

A significant difference in general health was observed in the presence of comorbid chronic illnesses (*p* < 0.01). Patients with cardiac disease and diabetes mellitus, coupled with hypertension, were in much worse general health. Young patients also feel more of an emotional burden compared to older patients. Also, patients with 5–8 mg/l levels of serum creatinine had lower emotional wellbeing. The low-income groups were also affected regarding social function. All the variables used in this study to measure the QoL had a positive correlation with the highest burden found in physical activities. In contrast, the lowest was found in social function.

The study further reveals that these patients' physical function is 45.666 (95% CI, 44.291, 47.041), which is less than half of the function of a normal human being, which is 100. This shows that the burden of the disease seems very severe. This study's mean emotional QoL score was 53.33 (95% CI, 51.1, 55.5), which is only slightly more than half of the QoL score in a healthy human.

The same findings have been revealed in a multicenter study among dialysis patients in India. Even after interventions, the patients revealed lesser physical QoL than emotional QoL, which was statically significant [19, 20]. In 2017, a study was conducted in Kuwait in the QoL among 336 hemodialysis patients. The study concluded the same findings as the current study that there must be a strengthening of emotional support to patients on hemodialysis to further improve their QoL [21]. In 2012, a descriptive cross-sectional study was conducted in Iran on the QoL of 46 hemodialysis and 46 P.D. patients. The study concluded that the leading underlying causes of kidney dysfunction were diabetes and hypertension. Compared to physical QoL, emotional QoL was reduced among hemodialysis patients [22].

The same findings have also been found in European countries. In 2015, a study in Greece corroborated the findings of the current study. The patients with cardiac disease and underweight patients suffered physical functioning (*p* < 0.05). Young people, educated people, widowed people, and people on low-income group had reduced quality of life (*p* < 0.05). The educated groups were much more affected than the noneducated or primary educated group in their physical activity (*p* < 0.02). A significant difference in general health was also observed in the presence of comorbid chronic illnesses (*p* < 0.01). Patients with cardiac disease and diabetes mellitus with hypertension illness had much worse general health scores. Studies conducted around the globe are supporting the same findings. Older adults have lower expectations, which in turn reduces their motivation to do physical activity. At the same time, younger adults are motivated to do more activity and experience more fatigue, which results in poor physical QoL [23]. Various social and clinical parameters had influenced the emotional dimensions of QoL among dialysis patients in this study. Young patients feel more emotionally fragile. Patients with 5–8 mg/l level of serum creatinine had less emotional wellbeing. The low-income groups were also significantly affected by social function (*p* < 0.01). Similar findings have been shown by Gerasimoula [23].

In 2016 in Pittsburgh, the United States, a group of researchers revealed that patients undergoing hemodialysis for more than six months experienced reduced functional status, more hospital readmissions, and a lower quality of life, which was statistically associated with severe depression. They were more emotionally unstable due to poor QoL related to depression [24]. QoL and fatigue had a negative relationship with anxiety and depression and positive correlation with health status [23].

There was a significant effect of the patient's QoL physical functioning with older age (*p* < 0.05), income (*p* < 0.04), and frequency of dialysis (*pp* < 0.03). There was a higher significance of patent's QoL role physical with education (*p* < 0.01) and diabetes mellitus (*p* < 0.04). Other studies showed that QoL of patients undergoing hemodialysis was impaired and affected by age, sex, occupation, marital status, type of work, socioeconomic status, residence, and educational level [25].

There is a positive relationship between patient's pain with older age (*p* < 0.03) and frequency of dialysis (*p* < 0.05). There is positive QoL of general health with age (*p* < 0.01), diabetes mellitus (*p* < 0.02), and frequency of dialysis (*p* < 0.03). There was significant relationship between QoL fatigue and age (*p* < 0.05), education (*p* < 0.03), and duration of dialysis (*p* < 0.04). Other studies showed that all domains of QOL like physical, psychological, social relationships, and environment were affected [26]. QoL increased with acceptance of illness and was correlated with physical, psychological, and environmental domains [27].

The QoL score decreased with increasing age, lower education, lower income, diabetes mellitus, increased duration of dialysis, increased frequency of dialysis, increased serum creatinine, and increased body mass index. QoL physical functioning, role physical, pain, general health, and fatigue affected the determinants of demographic and clinical variables. Other studies show that age and gender significantly affected the QoL score in patients on dialysis [28]. There was a negative association between health related QoL with age, comorbid illness, and medications [29, 30]. Low KDQOL scores were observed for the symptoms, effects, burden, work status, and sleep [31]. Higher physical, mental component summary, and total KDWOL scores were associated with higher education, low comorbidity, low body mass index, and higher GFR, and lower HbA1c [31]. This study also shows physical and emotional QoL scores in dialysis patients are lower, and they have an overall poor QoL.

## 12. Limitation

The study was conducted in only one setting. Hence, larger patients' participation from various centers of various geographical locations is needed. We used a cross-sectional design and did not identify a causal relationship between the variables. We used convenience sampling which may have resulted in bias interpretation of the results and conclusions. We could have explored the missed hemodialysis treatments, predictors, and outcomes among the hemodialysis population.

## 13. Conclusion

Our study showed there was a significant effect of the patient's QoL physical functioning with older age (*p* < 0.05), income (*p* < 0.04), and frequency of dialysis (*p* < 0.03). There was a higher significance of patent's QoL role physical with education (*p* < 0.01) and diabetes mellitus (*p* < 0.04). There is a positive relationship between patient's pain with older age (*p* < 0.03) and frequency of dialysis (*p* < 0.05). There is positive QoL of general health with age (*p* < 0.01), diabetes mellitus (*p* < 0.02), and frequency of dialysis (*p* < 0.03). There was significant relationship between QoL fatigue and age (*p* < 0.05), education (*p* < 0.03), and duration of dialysis (*p* < 0.04). Elderly patients, lower income, and increased frequency of dialysis had poor QoL for physical functioning. Low education and diabetes mellitus had poor QoL for role physical. Elderly patients and increased frequency of dialysis per week had lower QoL for pain and general health. Elderly patients, lower education, and longer duration of dialysis were associated with poor QoL for fatigue. The treatment with hemodialysis in patients is important in improving QoL. The results of the study provide evidence for Ministry of health, medical professionals, and clinical nurses to prioritize healthcare and effective treatment plans. Both physical and emotional QoL scores in Omani dialysis patients are nearly half those of a normal human. The study has revealed that there is a poor QoL among dialysis patients like other studies. Hence, the report can support planning the priorities and clinical practice guidelines for best practices in renal rehabilitation in dialysis centers to improve patients' QoL.

## Figures and Tables

**Table 1:** Mean of the score of the measures of emotional quality of life.

Measures	Mean scores	Standard deviation
Role-emotional	56.43	17.73
Emotional well	45.37	35.95
Social function	58.21	24.31

**Table 2:** Kidney Disease Quality of Life Scale, definition, and its items.

No	Scale	Number of items	Definition of scale
1	Physical functioning–(PF)	10	Limitations in physical activity because of health problems
2	Social functioning (SF)	2	Limitations in social activities because of physical or emotional problems
3	Role limitations–physical (RP)	4	Limitations in usual role activities because of physical health problem
4	Bodily pain (BP)	2	Presence of pain and limitations due to pain
5	General medical health (GH)	5	Self-evaluation of personal health
6	Mental health (MH)	5	Psychological distress and well-being
7	Role limitations–emotional (RE)	3	Limitations in usual role activities because of emotional problems
8	Vitality (VT)	4	Energy and fatigue
9	General health perceptions	1	

**Table 3:** Mean of the score of the measures of physical quality of life.

Measures	Mean scores	Standard deviation
Physical functioning	41.1	20.12
Role physical	38.54	31.13
Pain	52.6	25.2
General health	49.15	10.62
Fatigue	46.95	16.48

**Table 4:** Factors of QoL and relationship with demographic and clinical variable.

Physical component	Age	Education	Income	Diabetes mellitus	Duration of dialysis	Frequency of dialysis	Serum creatinine	Body mass index
Physical functioning	0.05	0.8	0.04	0.83	0.45	0.03	0.76	0.80
Role physical	0.7	0.01	0.54	0.04	0.25	0.29	0.33	0.27
Pain	0.03	0.91	0.82	0.51	0.37	0.05	0.27	0.12
General health	0.01	0.83	0.33	0.02	0.67	0.03	0.11	0.96
Fatigue	0.05	0.03	0.34	0.67	0.04	0.64	0.84	0.20
Mental component								
Role-emotional	0.40	0.65	0.63	0.93	0.49	0.36	0.15	0.72
Emotional well	0.82	0.72	0.63	0.34	0.83	0.59	0.45	0.52
Social function	0.04	0.45	0.43	0.67	0.24	0.21	0.32	0.49
Vitality	0.26	0.85	0.36	0.21	0.75	0.38	0.47	0.95
Mental health	0.67	0.46	0.56	0.45	0.34	0.13	0.21	0.85

## Data Availability

Data used to support this study are available from the corresponding author upon reasonable request.
